# Gentiobiose and cellobiose content in fresh and fermenting cucumbers and utilization of such disaccharides by lactic acid bacteria in fermented cucumber juice medium

**DOI:** 10.1002/fsn3.1830

**Published:** 2020-09-25

**Authors:** Redife Aslihan Ucar, Ilenys M. Pérez‐Díaz, Lisa L. Dean

**Affiliations:** ^1^ Department of Food, Bioprocessing, and Nutrition Sciences North Carolina State University Raleigh NC USA; ^2^ Food Science & Market Quality and Handling Research Unit USDA‐Agricultural Research Service Raleigh NC USA; ^3^ Food Science & Market Quality and Handling Research Unit USDA‐Agricultural Research Service Raleigh NC USA

**Keywords:** cellobiose, cucumber fermentation, disaccharides, gentiobiose, LAB

## Abstract

The content of cellobiose and gentiobiose, cellulose‐derived dissacharides, in fresh and fermented cucumber was evaluated along with the ability of *Lactobacillus plantarum*, *Lactobacillus pentosus*, *Lactobacillus buchneri* and *Lactobacillus brevis* to utilize them during and after fermentation. The disaccharide content in fresh and fermenting cucumbers was below the detection level (10 µM) using HPLC for analysis. Utilization of cellobiose and gentiobiose by lactic acid bacteria (LAB) was tested in fermented cucumber juice medium (FCJM), a model system for the bioconversion and postfermentation lacking glucose and fructose. Changes in the fermentation metabolites were followed using HPLC and pH measurements as a function of time. The disaccharides were utilized by *L. plantarum*, *L. pentosus,* and *L. buchneri* in FCJM at pH 4.7 ± 0.1, representative of the active fermentation period, and converted to lactic acid. The disaccharides were not utilized in FCJM at pH 3.7 ± 0.1, representative of the end of fermentation. While *L. brevis* was unable to utilize cellobiose efficiently in FCJM, they were able to remove gentiobiose at pH 4.7 ± 0.1. Some strain level differences in cellobiose utilization were observed. It is concluded that the disaccharides are absent in the fresh cucumber and the typical fermentation. The LAB prevalent in the bioconversion utilizes cellobiose and gentiobiose, if available, at pH 4.7 ± 0.1. The LAB would not remove the disaccharides, which could become available from cellulose degradation by the acid resistant indigenous microbiota, after the pH is reduced to 3.7 ± 0.1.

## Graphical abstract

*Lactobacillus pentosus* and *Lactobacillus plantarum* can remove the disaccharides cellobiose and gentiobiose during a cucumber fermentation if the pH is above 3.7, enhancing the completion of the bioconversion. The disaccharides were not detected in fresh and fermented cucumbers.

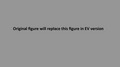

## 1 INTRODUCTION

Completion of a cucumber fermentation by lactic acid bacteria (LAB) is typically monitored by measuring the reduction of the sugars naturally present in the fruits, mainly glucose and fructose, and the formation of lactic acid, acetic acid, and ethanol, using high‐performance liquid chromatography (HPLC). The disappearance of all the glucose and fructose in fermentation vessels signals the end point of a cucumber fermentation. Industrially, measurements of pH with time are to follow the progress of a cucumber fermentation. Commercial production has capitalized on the use of reductive sugars strips, typically used for urinalysis, to routinely monitor the completion of fermentation in batches prior to processing. Despite the fact that HPLC analysis enables the concomitant detection of glucose, fructose, organic acids, and ethanol, expands the sensitivity threshold, and enables a determination of specific yields in cucumber fermentation, a comprehensive understanding of the metabolic bioconversions that impart quality attributes to specific batches is lacking (Lu, Fleming, & McFeeterss, 2002; McFeeters, 1993). Flavors vary among fermented batches as well as microbial stability during long‐term storage (Franco, Pérez‐Díaz, Johanningsmeier, & McFeeters, 2012).

Metabolic profiling of anaerobically fermented cucumbers that were spoiled by *Lactobacillus buchneri* revealed changes in 92 compounds including citrulline, trehalose, cellobiose, xylose, lyxose, gentiobiose, and lactic acid (Johanningsmeier & McFeeters, 2011, 2015). Incremental concentrations were observed in alcohols and butanoic and pentanoic acids and were accompanied by decreases in the concentration of monosaccharides, disaccharides, amino acids, nucleosides, long chain fatty acids, and ketones (Johanningsmeier & McFeeters, 2011, 2015). It was additionally observed that citrulline, d‐trehalose, and d‐cellobiose were particularly utilized by *L. buchneri* prior to lactic acid degradation (Johanningsmeier & McFeeters, 2015).

This study evaluated the function of disaccharides, particularly cellobiose and gentiobiose, as potential energy sources in cucumber fermentations for selected LAB. It was theorized that a greater understanding of the role of energy sources found in cucumber fermentations for LAB would enable the design of functional starter or adjunct cultures that are able to complete the bioconversion and remove secondary energy sources. In doing so, a starter/adjunct culture would be able to prevent the growth of spoilage microbes during long‐term storage and postfermentation.

Cellobiose and gentiobiose are disaccharides composed of two glucose units joined by a β‐(1‐4) or β‐(1‐>6) glycosidic linkage, respectively. Both disaccharides are plant metabolites found in some food products such as vegetables, fruits, corn syrups, and others (Buckenhüskes, 1997). Cellobiose is the product of cellulose or a plant β‐glucan degradation. A number of microbes can breakdown cellobiose to the glucose subunits via β‐glucosidases (Abdel‐Rahman, Tashiro, Zendo, Shibata, & Sonomoto, 2011; Singhvi, Joshi, Adsul, Varma, & Gokhale, 2010). The metabolism of disaccharides by LAB is regulated via the phosphotransferase systems (PTS) and/or intracellular phospho‐glycosyl hydrolases (Andersen et al., 2012; Barrangou et al., 2006; Francl, Thongaram, & Miller, 2010). Various β‐glucosides are translocated by LAB via the PTS, and subsequently cleaved by phospho‐β‐glucosidase to produce phosphorylated glucose and the respective glycon (Aleksandrzak‐Piekarczyk, Polak, Jezierska, Renault, & Bardowski, 2011; Bardowski, Ehrlich, & Chopin, 1994, 1995; Schnetz, Toloczyki, & Rak, 1987; Tobisch, Glaser, Krüger, & Hecker, 1997). Specific PTS permeases and the 6‐phospho‐β‐glucosidase are regulated by cellobiose and gentiobiose in *Lactobacillus acidophilus* at the transcriptional level (Andersen et al., 2012). The cellobiose PTS permease coding gene in *L. acidophilus* is homologous to the functionally characterized system in *Lactobacillus gasseri* (Andersen et al., 2012). The cellobiose and gentiobiose induced PTSs form a unique phylogenetic cluster among the *L. acidophilus* PTSs (Andersen et al., 2012). However, a gentiobiose‐specific PTS has not been functionally characterized in *L. gasseri* and/or other LAB (Andersen et al., 2012; Francl et al., 2010). A putative cellobiose PTS was found in *Lactobacillus paracasei* by comparative genomics (Smokvina et al., 2013). Such gene cassettes varied among *L. paracasei* strains (Smokvina et al., 2013). In *Lactococcus lactis,* the cellobiose‐specific PTS system is comprised of CelB, PtcB, and PtcA, is CcpA‐dependent and able to transport lactose (Aleksandrzak‐Piekarczyk et al., 2011).

While a number of lactobacilli, including *Lactobacillus plantarum*, *Lactobacillus johnsonii*, *Lactobacillus casei*, *L. gasseri*, *L. paracasei*, and *L. acidophilus*, have a diversity of disaccharide hydrolases or disaccharide‐phosphate hydrolases, some, such as *Lactobacillus brevis*, *Lactobacillus reuteri*, *L. buchneri*, *Lactobacillus spicheri*, and *Lactobacillus delbrueckii* are restricted in this regard (Andersen et al., 2012; Barrangou et al., 2006; Fitzsimons, Cogan, Condon, & Beresford, 1999; Francl et al., 2010; Gänzle & Follador, 2012; Rogosa, Franklin, & Perry, 1961; Yu et al., 2012). Differences in disaccharides utilization have also been observed at the strain level. 90% of a group of 72 *L. plantarum* isolates from vegetables and fruits were able to use d‐cellobiose, while less than 50% were capable of fermenting gentiobiose. Variability in the ability to utilize cellobiose and gentiobiose by 10 *L. plantarum* isolated from Thai fermented vegetables and fruits was also observed by Tanganurat, Quinquis, Leelawatcharamas, and Bolotin (2009). Lactobacilli able to utilize cellobiose convert it to l‐ and d‐lactic acid (Carr, Chill, & Maida, 2002; Fitzsimons et al., 1999; Gänzle, 2015; Mao, Chen, & Horvath, 2015; Soltan Dallal, Zamaniahari, Davoodabadi, Hosseini, & Rajabi, 2017; Tamang et al., 2005; Yu et al., 2012).

This study confirmed the concentration of cellobiose and gentiobiose naturally found in fresh and fermented cucumbers using HPLC analysis for quantification and evaluated the ability of selected LAB relevant to cucumber fermentations to utilize these sugars. A fermented cucumber juice (FCJ) model system was used to evaluate the ability of *L. plantarum*, *L. pentosus*, *L. buchneri*, *L. brevis*, and *P. pentosaceus* to utilize cellobiose and gentiobiose to simulate conditions during (pH 4.7) and after (pH 3.7) fermentation. Microbial growth and fermentation of cellobiose and gentiobiose were monitored by colony counts from deMan, Rogosa and Sharpe (MRS) agar plates and by pH and metabolite concentration measurements using a probe and HPLC analysis, respectively.

## 2 MATERIALS AND METHODS

### 2.1 Measurement of cellobiose and gentiobiose concentration in fresh and fermented cucumbers

Samples of four fresh, size 2B (3.18 cm to 3.79 cm in diameter), pickling cucumber lots to be fermented in commercial vessels were obtained from a local processor. The corresponding fermented cucumber samples were collected on days 3 and 38 of fermentation together with the fermentation cover brine in a 50:50 ratio. Fresh and fermented cucumbers were sliced using aseptic techniques and blended using a Waring Commercial Blender 700S (Torrington, CT, USA) equipped with a sterilized glass cup for 90 s at medium speed. Cucumber slurries were homogenized using a Seward Stomacher 400 (Bohemia, NY, USA) in 6″ × 4.5″ filter stomacher bags for 1 min at maximum speed. The slurries were subjected to two cycles of freezing and thawing to enable the release of all the sugars from the tissue into the liquid phase. One mL aliquots of the filtered homogenate were spun at 15,294 rcf for 10 min in an Eppendorf benchtop refrigerated centrifuge 5810R (Hamburg, Germany) to remove residual particulate matter. Supernatants were used for HPLC analysis to determine the cellobiose and gentiobiose concentrations.

Aliquots of 100 µl of fresh cucumber juice, juice extracted from cucumbers fermented in commercial vessels or from experimental media were diluted to 2 ml with water spiked with 50 µl of an internal standard of lactose (Sigma‐Aldrich). All solutions were filtered through Dionex OnGuard‐H cartridges (Dionex Corporation) to remove free amino acids into autosampler vials. The extracts were analyzed using a Dionex BioLC (Dionex Co.) at a controlled temperature of 25°C. The system consisted of a gradient pump, an autosampler, and a Pulsed Amperometric Detector. The mobile phase was 50 mM NaOH (Thermo Fisher Scientific) at an isocratic flow rate of 1.0 ml/min. The column used was PA‐1, 250 mm length and 4 mm i.d. (Dionex Co.), fitted with PA‐1 Guard column (Dionex Co.). The detector was programmed to run a quadruple waveform as recommended by the manufacturer. The detector sensitivity was set to 500 nCoulombs (nC).

The injection volume was 10 µl. Each sugar was quantified by calculating a ratio of the unknown peak height to the lactose (internal standard) peak height and comparing it with a ratio of a known concentration of cellobiose and gentiobiose (Sigma‐Aldrich and Fluka Chemie, Steinheim, Germany, respectively; Pattee, Isleib, Giesbrecht, & McFeeters, 2000).

### 2.2 Bioinformatic analysis of the genes coding for enzymes involved in cellobiose and gentiobiose metabolism by certain LAB

Detection of the cellobiose permease gene, *celB*, among the bacterial genomes sequenced to date was done using the Integrated Microbial Genomics, Find Gene function (Chen et al., 2016; https://img.jgi.doe.gov/cgi‐bin/m/main.cgi). The analysis of the putative enzymes involved in the metabolism of gentiobiose and cellobiose was conducted using the publicly available genome sequences for *L. pentosus* (3), *L. plantarum* (107), *L. brevis* (21), *L. buchneri* (6), and *P. pentosaceous* (7). The Joint Genome Institute‐Integrated Microbial Genomes platform (Chen et al., 2016) was used to develop informative metabolic maps with regard to the abundance of certain genes in the genomes described above. The KEGG Orthology Pathways (KO; Kanehisa, Sato, Kawashima, Furumichi, & Tanabe, 2016; Kanehisa, Sato, & Morishima, 2016; http://www.kegg.jp/kegg/pathway.html) tool was specifically used to populate Table 2. The Metacyc (Caspi et al., 2017) and Biocyc (Karp et al., 2017) online tools at the IMG platform were used to define and/or confirmed the reference pathways for glycolysis and β‐glucosidases involved in cellobiose and gentiobiose catabolism.

### 2.3 Preparation of fermented cucumber juice media (FCJM)

Size 2B (32–38 mm in diameter) fresh whole pickling cucumbers from two different lots were secured from the local retail (Raleigh, NC, USA). Fresh pickling cucumbers in good condition and free of mechanical damage were selected, washed with plain water, and packed into four one‐gallon glass jars, two jars for each cucumber lot, using a 50:50 (w/v, cucumbers/cover brine) pack‐out ratio. The cover brine was prepared so that it equilibrated with the cucumbers at 80 mM CaCl_2_ (Brenntag), 6 mM potassium sorbate (Mitsubishi International Food Ingredients), 10.1 mM Ca(OH)_2_ (Sigma‐Aldrich), and 44 mM acetic acid, added as 20% vinegar (Fleischmann Vinegar), to adjust the initial pH to 4.7 ± 0.1. A mixed starter culture of *L. plantarum* 3.8.2 and *L. pentosus* LA 0445 (previously identified as *L. plantarum* LA0445; McDonald, Shieh, Fleming, McFeeters, & Thompson, 1993; Table 1) was prepared as described below and supplemented to 10^5^ CFU/ml. Jars were closed with commercial metal lug caps that were heated in boiling water for 10 s to soften the plastisol liner. Each lid was equipped with a rubber septum in its center to allow for sampling of cover brine using a 10‐ml syringe attached to a 18G × 1 1/2″ needle (Becton Dickinson Co.). The jars were incubated at 30°C for 10 days. The pH was measured using a Fisher Accumet pH meter (Model AR25, Fisher Scientific) combined with a Gel‐Filled Pencil‐Thin pH Combination Electrode (Acumet, Fisher Scientific). The completion of the fermentation was confirmed by measuring sugars, organic acids, and ethanol in cover brine samples using the HPLC analysis conducted as described below. At the end of these fermentations, the pH was determined to be 3.3 ± 0.1 and the media contained 0.5 ± 0.2 mM and 1.69 ± 0.6 mM glucose and fructose, respectively.

**TABLE 1:** Description of the lactic acid bacteria strains used in this study

Genus	Species	ID number(s)	Sources	Reference
*Lactobacillus*	*plantarum*	LA0070; ATCC 14917	Pickle cabbage	ATCC < PA Hansen < Roy Techn. Coll., Copenhagen < S. Orla‐Jensen (*Streptobacterium plantarum*) https://www.atcc.org/products/all/14917.aspx#history
*Lactobacillus*	*plantarum*	LA1196; ATCC BAA‐793; NCIMB 8826; WCFS1	Saliva	Hols et al. (1997)
*Lactobacillus*	*plantarum*	3.2.8	Commercial cucumber fermentation	Peréz‐Díaz et al. (2016)
*Lactobacillus*	*pentosus*	LA0233; ATCC 8041	Sauerkraut	Fred et al. (1921)
*Lactobacillus*	*pentosus*	LA0445; BI0007, MOP3	Commercial cucumber fermentation	Fleming, McFeeters, Daeschel, Humphries, and Thompson (1988)
*Lactobacillus*	*pentosus*	1.8.9	Commercial cucumber fermentation	Peréz‐Díaz et al. (2016)
*Lactobacillus*	*brevis*	LA0200; ATCC 8287	Green Sevillano fermenting olives	RH Vaughn 269Y; Dunn, Shankman, Camien, and Block (1947)
*Lactobacillus*	*brevis*	LA0036; ATCC 14869, NRRL B‐4527	Human feces	ATCC < PA Hansen < Roy. Techn. Coll., Copenhagen < S. Orla‐Jensen 14 (*Betabacterium breve*); Rogosa & Hansen, 1971
*Lactobacillus*	*brevis*	7.2.43	Commercial cucumber fermentation	Peréz‐Díaz et al. (2016)
*Lactobacillus*	*buchneri*	LA0030; ATCC 4005, NRRL B1837	Tomato pulp	Rogosa and Hansen (1971)
*Lactobacillus*	*buchneri*	LA1149	Commercial cucumber fermentation	Franco et al. (2012)
*Lactobacillus*	*buchneri*	LA1147; E‐33‐07	Commercial cucumber fermentation	Franco et al. (2012)

The cover brine and juice from fermented cucumbers was used to prepare FCJM. Fermentation cover brines were decanted from the jars into 2‐L beakers. The fermented cucumbers were passed through an automatic juice extractor (Juiceman Jr. Model JM‐1, Beachwood, Ohio, USA) to separate the pulp from the liquid content on a jar volume basis. The pulp remaining in the fermented cucumber juice was removed by straining with a 100% cotton cheesecloth (grade #90, 44 × 36 threads/inch, Cartridge Plus, Inc., Riva, MD, USA) and a subsequent centrifugation at 3,750 × *g* for 15 min at ambient temperature using a bucket rotor (Eppendorf Centrifuge Model 5810, Hamburg, Germany). The respective clear fermented cucumber juices and fermentation cover brines were mixed to the same ratio as in the fermentation jars to make up the FCJM. The FCJM derived from each cucumber lot were independently used and supplemented with gentiobiose (Sigma‐Aldrich G‐3000, 85% purity) or cellobiose (98% purity) as needed. The pH of the supplemented and un‐supplemented FCJM was adjusted to 4.7 ± 0.1 or 3.7 ± 0.1 as needed using a 5N NaOH solution (Spectrum Chemicals, NJ, USA) and 3N HCl (Spectrum Chemicals). The pH‐adjusted FCJM were filter sterilized using 0.2‐µ filtration units (Nalgene^®^‐Rapid Flow™, Thermo Scientific). Aliquots of 10 ml of each FCJM were aseptically transferred to 15‐mL conical tubes for experimentation.

### 2.4 LAB cultures preparation

The bacterial cultures used for experimentation are described in Table 1. The LAB cultures were transferred from frozen stocks, prepared with Lactobacilli MRS broth (Becton Dickinson Co.) supplemented with 15% glycerol (Sigma‐Aldrich), to 10 ml of MRS broth. The cultures were incubated at 30°C for 48 to 72 hr prior to inoculating fermentations or FCJM. The *L. plantarum* and *L. pentosus* cultures were transferred to fresh cucumber juice after growing in MRS broth and prior to inoculating the FCJM for the experiment designed to evaluate their ability to use cellobiose and gentiobiose under aerobiosis and anaerobiosis at different initial pH values. Fresh cucumber juice was prepared in the same way the fermented cucumber juice was prepared (as described above). The fresh cucumber juice was also filtered‐sterilized prior to inoculation. The FCJM was inoculated to 10^5^ CFU/ml. The optical density at 600 nm of the MRS or fresh cucumber juice cultures was measured using a Novaspec II, (Pharmacia, Stockholm, Sweden) and used to adjust the inoculation level. A sterile 0.85% NaCl (Sigma‐Aldrich) solution was used to adjust the inocula concentration as needed. Inocula with mixed cultures was prepared by combining the cells suspension in saline solution so that each strain will be at 10^5^ CFU/mL in the FCJM.

### 2.5 Evaluation of the ability of certain LAB to utilize gentiobiose and cellobiose in FCJM

The experimental design included the testing of the ability of three strains of *L. pentosus*, *L. plantarum*, *L. brevis* or *L. buchneri* (Table 1) to utilize cellobiose and gentiobiose in FCJM under aerobiosis and at an initial pH of 4.7. Thus, there were two treatments (FCJM with cellobiose and FCJM with gentiobiose) for each mono‐species‐mixed culture tested, consisting of three strains per species, with technical and independent duplicates for a combined total of four replicates per species and treatment. Additionally, the mixed cultures were inoculated in the un‐supplemented FCJM. All FCJM cultures were incubated at 30°C for 7 days to enable secondary fermentation.

Samples were aseptically collected at the end point of incubation and were serially diluted in a sterile 0.85% NaCl solution prior to spiral plating on Lactobacilli MRS media using an Autoplate 4000 Eddy Jet 2 spiral plater (IUL, Barcelona, Spain). MRS agar plates were incubated at 30°C aerobically for 48 hr. Colony counts from MRS agar plates were obtained using a Flash & Go Automatic Colony Counter (IUL). The detection limit for colony counts was 2.4 log CFU/ml.

The pH and fermentation biochemistry were monitored at the end point only. Changes in pH were monitored as described above. The fermentation biochemistry was monitored by HPLC analysis of FCJM samples collected aseptically from the 15‐ml conical tubes containing the cultures. 1.5 ml of each fermentation sample was spun at 15,294 × *g* for 15 min at room temperature using an Eppendorf Centrifuge Model 5810 (Hamburg, Germany). A minimum of 500 µl of the supernatants were transferred into glass HPLC vials. Organic acids and carbohydrate concentrations were measured using a 30‐cm HPX‐87H column (Bio‐Rad Laboratories) and the HPLC method described by McFeeters (1993) with some modifications. The operating conditions of the UFLC Shimadzu HPLC (Shimadzu Corporation) system were a column temperature of 65°C and a 0.01 N H_2_SO_4_ eluent at 0.9 ml/min. The diode array detector was set at 210 nm at a rate of 1 Hz to quantify malic, lactic, succinic, propionic, and butyric acids. An RID‐10A refractive index detector (Shimadzu Corporation) connected in series with the diode array detector was used to measure acetic acid, lactic acid, glucose, fructose, and ethanol. External curves were also run using at least five concentrations of glucose, fructose, lactic acid, acetic acid, and ethanol for quantification purposes.

### 2.6 Evaluation of the ability of single strains of *L. pentosus* and *L. plantarum* to utilize gentiobiose and cellobiose in FCJM under aerobiosis and anaerobiosis

The experimental design included the testing of the ability of three individual strains of *L. pentosus*, including ATCC8041, LA0445 and 1.8.9 and three individual strains of *L. plantarum*, particularly ATCC14917, WCSF1 and 3.2.8 (Table 1), to utilize cellobiose and gentiobiose in FCJM under aerobiosis and anaerobiosis at an initial pH of 4.7 or 3.7. Thus, there were six treatments for each strain tested with technical and independent duplicates for a combined total of four replicates per strain and treatment of 48 samples. This experimental design generated a total of 96 independent experimental tests plus twelve noninoculated FCJM controls (2 per media type, for each lot). Additionally, the cultures were inoculated in the un‐supplemented FCJM. All FCJM cultures were incubated at 30°C for 7 days to enable secondary fermentation. Samples were aseptically collected at the end point of incubation and plated on Lactobacilli MRS agar plates to determine colony counts as described above. The pH and fermentation biochemistry were monitored at the end point as described above.

## 3 RESULTS AND DISCUSSION

### 3.1 Cellobiose and gentiobiose concentration in fresh and fermented cucumber samples

The potential role of cellobiose and gentiobiose as energy sources in cucumber fermentation for certain LAB was defined. It was found that although cellobiose and gentiobiose had been detected in cucumber fermentation spoilage samples using two‐dimensional gas chromatography time‐of‐flight‐mass spectrometry (GC × GC‐TFMS; Johanningsmeier & McFeeters, 2015), concentrations below the limit of detection were found in fresh and fermented cucumber samples using HPLC analysis (<10 µM). Four fresh cucumber samples and seven fermented cucumber samples were included in the HPLC analysis. The seven fermented cucumber samples consisted of triplicates from 3‐day‐old fermentations and quadruplets from 38‐day‐old fermentations. The marked differences in the results is explained by either the sensitivity of the two techniques or fluctuations in the disaccharides content as a function of the fresh cucumber physiological state, not considered in either study, or the fermentation stage. Our results do suggest that the lack of the disaccharides in the fresh fruits results in their absence in the fermentation and is thus not formed as the result of the primary bioconversion. Additional experimentation is needed to determine whether the disaccharides become available as energy sources in a prespoilage stage postfermentation and/or as the result of the metabolism of cellulose by an atypical fermentation microbiota.

### 3.2 Scrutiny of the putative pathways for cellobiose and gentiobiose utilization by lactic acid bacteria using a bioinformatic analysis

A putative *celB* was not found in *L. plantarum*, *L.pentosus*, *P. pentosaceous* and *L. brevis*. However, the putative gene coding for *celB*, the cellobiose‐specific permease EIIC component, was found in three *Lactococcus lactis* subspecies including *cremoris*, *lactis,* and *hordniae*, six strains of *Lactobacillus salivarius*, *Lactobacillus helveticus* DSM 20075, *Lactobacillus kefiranofaciens* ZW3, 11 strains of *L. acidophilus* (as expected), *L. buchneri* VBLLa 18‐02, and CD034 and *Pediococcus claussenii* TMW 2.54 and ATCC BAA‐344.

The enzymes coding for cellobiase was found by gene homology in most of the LAB genomes scrutinized, except for *Pediococcus pentosaceous* (Table 2). The presence of putative cellobiase in the *L. plantarum* genomes was strain dependent. *L. brevis* and *L. buchneri* had some glycolysis pathway‐related enzymes missing (Table 2).

**TABLE 2:** Bioinformatic analysis of the cellobiose and gentiobiose putative pathways in certain lactic acid bacteria of relevance to cucumber fermentations

Enzyme name	EC No.	Metabolic pathway	Target	Expected product	Species analyzed						
					*L. plantarum*	*L. pentosus*		*L. brevis*		*L. buchneri*	
Cellobiase	3.2.1.21	Starch and Sucrose	Cellobiose	β‐d‐glucose							
Phosphotransferase	2.7.1.69	Glycolysis	Cellobiose and Glucose	Lactic acid							
Phosphoglucomutase	5.4.2.2										
Glucokinase	2.7.1.2										
Aldose 1‐Epimerase	5.1.3.3										
Glucose‐6‐Phosphate Isomerase	5.3.1.9										
6‐Phosphofructokinase	2.7.1.11										
Hexose Diphosphatase	3.1.3.11										
Aldolase	4.1.2.13										
Triose‐Phosphate Isomerase	5.3.1.1										
Glyceraldehyde‐3‐Phosphatase	1.2.1.12										
Phosphoglycerate Kinase	2.7.2.3										
Phosphoglycerate Mutase	5.4.2.11										
Phosphoglycerate Mutase	5.4.2.12										
Phosphopyruvate Hydratase	4.2.1.11										
Pyruvate Kinase	2.7.1.40										

Note

The colored boxes mark the % of strains coding for a specific putative enzyme where red, green, and yellow represent more than 97%, less than 5% and missing genes, respectively.

### 3.3 Ability of certain LAB to utilize cellobiose and gentiobiose under aerobiosis at an initial pH of 4.7 ± 0.1

**Figure 1:**
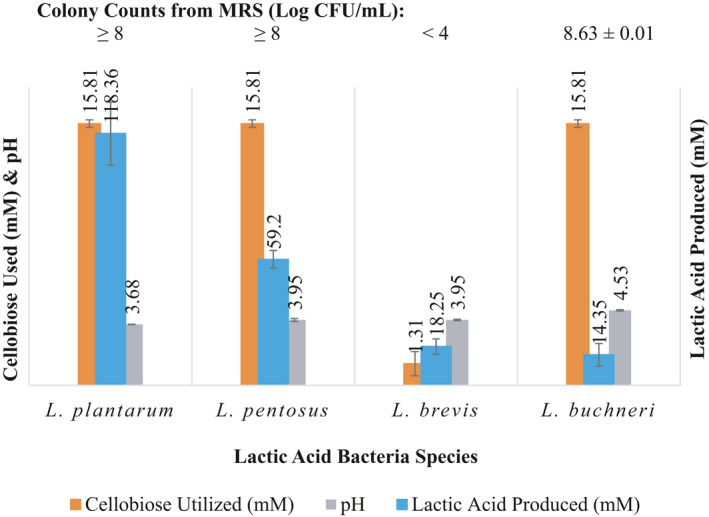
Disappearance of cellobiose from FCJM at pH of 4.7 ± 0.1 inoculated with lactic acid bacteria. The colony counts from MRS, pH, and lactic acid amounts formed are also shown. Acetate and ethanol were not detected in culture supernatants

**Figure 2:**
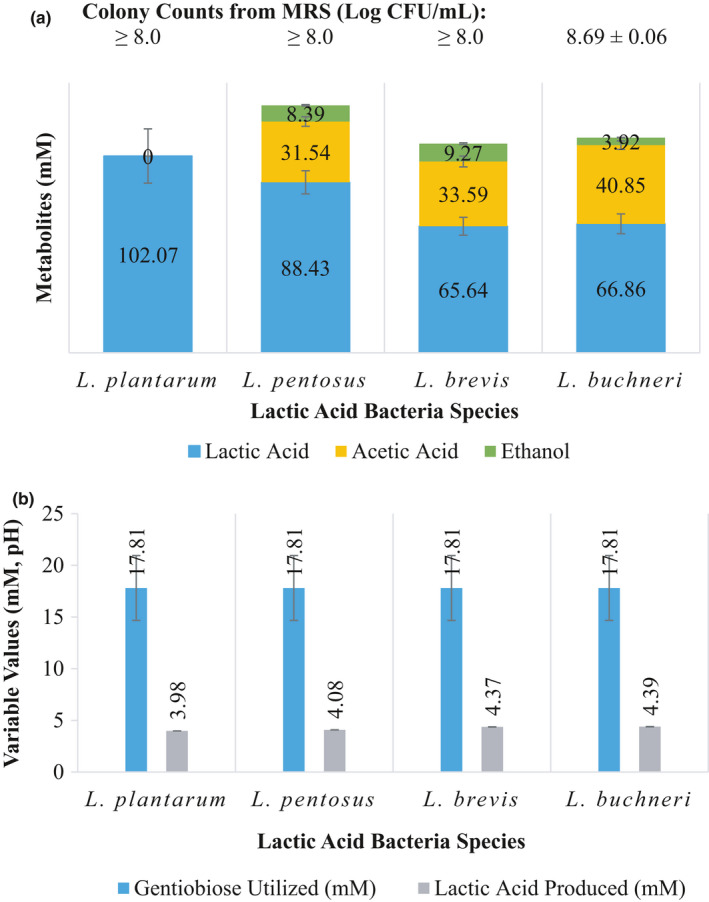
Disappearance of gentiobiose from FCJM inoculated with lactic acid bacteria. The FCJM pH was 4.7 ± 0.1. It is estimated that the gentiobiose source utilized contained at least 3 mM of the α‐anomer, melibiose. The colony counts from MRS (a), pH (a), and metabolites (b) are also shown

Figures 1 and 2 show the ability of LAB of relevance in cucumber fermentations to utilize the disaccharides, cellobiose and gentiobiose, in FCJM at pH 4.7 ± 0.1. As expected and in agreement with previous publications, *L. brevis* was unable to utilize cellobiose to a significant extent (Figure 1). *L. plantarum*, *L. pentosus,* and *L. buchneri* utilized most of the cellobiose added to the FCJM homofermentatively (Figure 1). *L. plantarum* produced an excess of lactic acid (Figure 1), which is speculated to have been derived from the residual energy sources in the FCJM such as glucose, fructose, and malic acid. For instance, the *L. plantarum* strains produced 46.53 ± 16.95 mM lactic acid in the un‐supplemented FCJM after 7 days of incubation under aerobiosis (data not shown). As expected, *L. pentosus* converted 15.81 ± 0.23 mM cellobiose to 59.20 ± 4.16 mM lactic acid after 7 days of incubation (Figure 1). The ability of *L. plantarum* and *L. pentosus* to utilize cellobiose resulted in a 3 log of CFU/mL increase in colony counts and a final pH of 3.8 ± 0.3, a 1 pH unit decrease (Figure 1). The conversion of cellobiose to lactic acid by *L. buchneri* was incomplete, presumably due to a slower acid production rate or the diversion of the carbons to other products, such as propanediol, that were not measured (Figure 1; Johanningsmeier & McFeeters, 2013). A slower acid production is also evidenced by the higher pH (4.53 ± 0.04) after 7 days of incubation, as compared to the other LAB cultures (Figure 1).

All the strains tested were able to utilize gentiobiose (Figure 2a). Except for *L. plantarum*, the lactobacilli converted gentiobiose into lactic acid, acetic acid, and ethanol in close to a heterofermentative ratio of 2:1:0.5 (Figure 2b). *L. plantarum* converted 17.81 ± 3.14 mM gentiobiose to 102.07 ± 14.07 mM lactic acid (Figure 2b). The ability to utilize the disaccharides resulted into microbial growth in the FCJM as determined by plating on MRS agar plates (Figure 2a). Changes in pH at the end point fluctuated between 0.4 and 0.7 pH units (Figure 2a).

Inclusion of the gentiobiose anomeric form, melibiose, in this study, was an unintended consequence associated with the use of the particular commercial preparation. The gentiobiose commercial preparation contained 15% melibiose as an impurity (~1.5 to 3 mM in the FCJM). Given the magnitude of the products concentration as the result of gentiobiose utilization, it is speculated that melibiose was also utilized by the LAB tested. Melibiose is rarely present in nature (Gänzle & Follador, 2012). However, transport systems for melibiose are suspected in *L. plantarum* (Tamura & Matsushita, 1992).

While the utilization of gentiobiose by the LAB of interest in cucumber fermentations, such as *L. brevis*, and *L. buchneri*, was observed in FCJM at pH 4.7 ± 0.1 (Figure 2), *L. brevis* was unable to utilize cellobiose as an energy source (Figure 1). *L. brevis* is a competitor of *L. pentosus* and *L. plantarum* in cucumber fermentations but grow relatively slower (Peréz‐Díaz et al., 2016). Perhaps the lack of competitiveness of *L. brevis* in cucumber fermentations is associated with a restricted energy source utilization profile. These observations are in agreement with those made by others documenting the inability of *L. brevis*, *L. buchneri*, *L. spicheri*, some leuconostocs, and *Enterococcus thailandicus* to utilize cellobiose (Carr et al., 2002; Hammes & Hertel, 2015; Mao et al., 2015; Sterr, Weiss, & Schmidt, 2009; Tamang et al., 2005). A cellobiose permease putative gene was not found in the LAB of interest except for *L. buchneri*. Contrary to *L. brevis*, *L. buchneri* has been associated with spoilage of fermented cucumbers (Franco et al., 2012; Johanningsmeier & McFeeters, 2013). The fact that *L. buchneri*, a spoilage microbe in cucumber fermentations, is able to utilize both of the disaccharides tested confirms the need to design starter or adjunct cultures that are able to remove energy sources other than glucose and fructose.

The LAB used in this study were able to utilize gentiobiose and cellobiose in FCJM suggesting that there are enough of other growth factors, such as amino acids, nucleosides, minerals, etc., in the fermented cucumber juice to sustain microbial proliferation. Furthermore, growth of *L. plantarum* and *L. pentosus* in the FCJM at pH 4.7 ± 0.1 was observed in the absence of added substrates. Two parameters were modified in the FCJM to enable microbial growth which were pH and the supplementation with an energy source. These observations confirm that the potential for spoilage in a given cucumber fermentation batch could be assessed by inoculating certain spoilage organisms in the corresponding FCJM as proposed by Fleming, McFeeters, and Thompson (1983). This approach represents a tool that could prevent important economic losses at the industrial scale production. More relevant is the understanding that batches of fermented cucumbers are primarily stable during long‐term storage due to the development of an extremely acidic pH and to a lesser extent due to the lack of readily available energy sources. Both of these parameters can change as a function of microbial metabolism or enzymatic activities.

### 3.4 Ability of three strains of *L. pentosus* and *L. plantarum* to utilize cellobiose and gentiobiose under aerobiosis and anaerobiosis at an initial pH of 4.7 and 3.7

**Figure 3:**
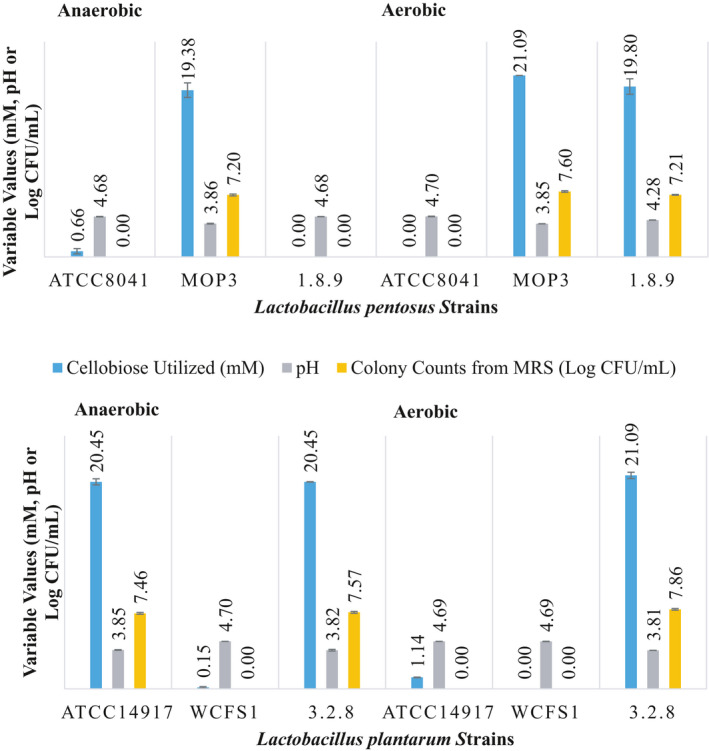
Utilization of cellobiose by single strains of *L. pentosus* and *L. plantarum* in FCJM at pH 4.7 under aerobiosis and anaerobiosis. The amount of cellobiose supplemented was 21.01 ± 0.08 mM. The colony counts from MRS and pH of FCJM at the end point are also presented

None of the disaccharides were utilized by the six strains of *L. plantarum* and *L. pentosus* scrutinized when the initial pH was adjusted to 3.7 ± 0.1. This observation is supported by the lack of a significant change in pH (ANOVA test at a *p* > .05), a reduction in colony counts to below detection limits and no changes in the concentrations of cellobiose and gentiobiose supplemented in the FCJM (data not shown). On the contrary, both bacterial species utilized the disaccharides under aerobiosis or anaerobiosis when the pH of the FCJM was adjusted to 4.7 ± 0.1 (Figures 3 and 5). Although the utilization of the disaccharides by *L. plantarum* and *L. pentosus* in FCJM at pH 3.7 ± 0.1 could occur during a prolonged incubation, β‐glucosidases are inhibited at pH below 4.0 (Kim, Lee, & Ma, 2017; Takase & Horikoshi, 1988; Yeoman et al., 2010; Zhong et al., 2016). While β‐glucosidases occur in many organisms, the activity of the enzymes derived from thermophilic bacteria and lactobacilli is known to be severely compromised at a pH of 4.0 with a 20% enzyme stability, as compared to a 40% stability at pH 5.0 (Kim et al., 2017; Takase & Horikoshi, 1988; Yeoman et al., 2010; Zhong et al., 2016). Growth of *L. plantarum* is known to stop at a pH of 3.3 with the cessation of acid production at a pH of 3.0 (McDonald, Fleming, & Hassan, 1990). Thus, a more reasonable interpretation is that *L. pentosus* and *L. plantarum* failed to utilize cellobiose and gentiobiose in FCJM at pH 3.7 given the lack of a β‐glucosidase activity.

**Figure 4:**
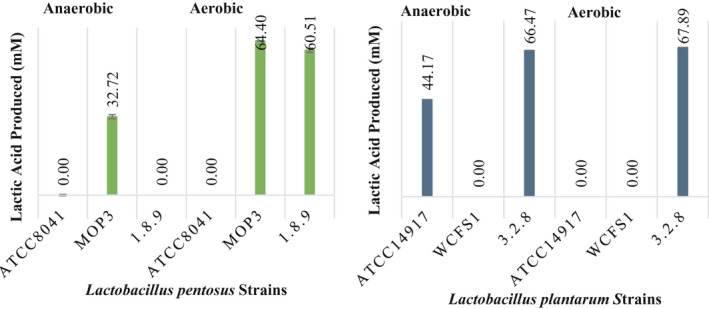
Production of lactic acid from cellobiose by single strains of *L. pentosus* and *L. plantarum* in FCJM at pH 4.7, under aerobiosis and anaerobiosis. The amount of cellobiose supplemented was 21.01 ± 0.08 mM

While the strains isolated from cucumber or cabbage fermentations were able to utilize cellobiose under aerobiosis or anaerobiosis in FCJM at pH 4.7 ± 0.1, strains of *L. plantarum* (WCSF1) isolated from saliva and *L. pentosus* (ATCC8041) obtained from sauerkraut (Fred, Peterson, & Davenport, 1921) were not able to utilize cellobiose in the same medium under either condition of oxygen availability (Figures 3 and 4). Some variations in the ability to utilize the disaccharides under aerobiosis and anaerobiosis were observed among the strains isolated from cabbage or cucumber fermentations (Figure 3). *L. plantarum* 3.2.8 and *L. pentosus* MOP3, both robust strains in cucumber fermentations (Anekella & Pérez‐Díaz, 2020), utilized cellobiose in FCJM at pH 4.7 ± 0.1 under aerobiosis and anaerobiosis. However, *L. pentosus* 1.8.9 isolated from commercial cucumber fermentations and *L. plantarum* ATCC14917 obtained from pickled cabbage were not able to utilize cellobiose in the same medium under anaerobiosis and aerobiosis, respectively (Figure 3).

Cellobiose and gentiobiose were converted homofermentatively and heterofermentaively, respectively, by certain *L. plantarum* and *L. pentosus* strains. The FCJM supplemented with cellobiose and gentiobiose carried out 98.00 ± 7.81 and 101.35 ± 9.07 mM lactic acid, respectively, from the primary fermentation. Additionally, there were 43.11 ± 3.89 mM acetic acid in the FCJM that was added to adjust the initial fresh cucumber fermentation pH to 4.7 ± 0.1. Residual ethanol in the amount of 11.73 ± 4.20 and 8.10 ± 2.71 mM was also present in the FCJM prior to the supplementation with the disaccharides. It was found that *L. plantarum* converted 21.01 ± 0.08 mM cellobiose to 66.18 ± 1.88 mM lactic acid in the FCJM at pH 4.7 ± 0.1 (Figure 4), suggesting the partial conversion of the cellobiose‐derived carbons to lactic acid. Similarly, *L. pentosus* converted cellobiose to 62.54 ± 1.95 mM lactic acid in FCJM at pH 4.7 (Figure 4). Residual fructose was not removed from FCJM when it was supplemented with cellobiose (data not shown). Changes in the FCJM pH of 0.86 log units resulted in pH values of 3.84 ± 0.02. Utilization of cellobiose resulted in at least a 2 log increase in cell density (Figure 3). A reduction in the cell concentration of the strains unable to utilize cellobiose was observed in FCJM at pH 4.7 ± 0.1 (Figure 3).

**Figure 5:**
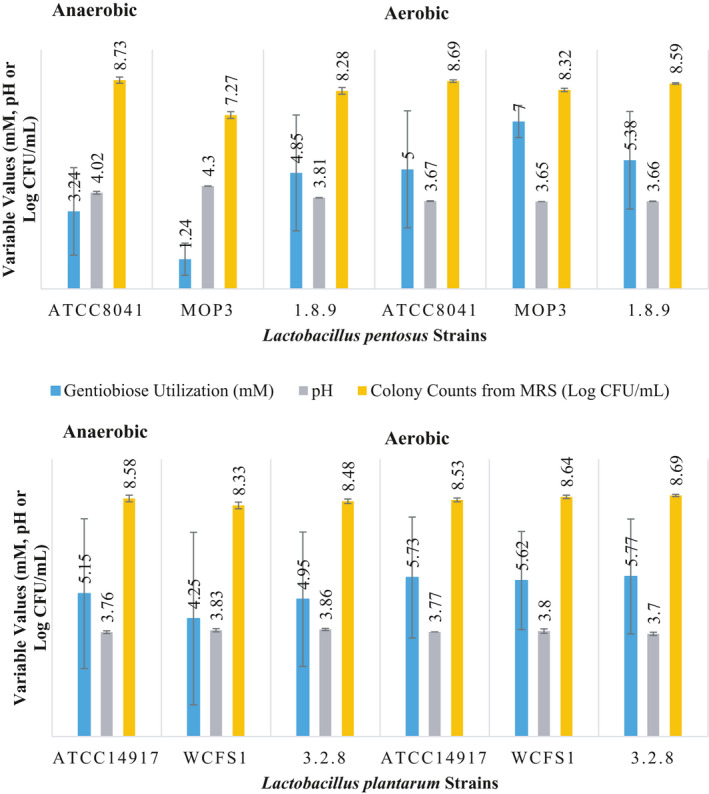
Utilization of gentiobiose by single strains of Lactobacillus pentosus and *Lactobacillus plantarum* in FCJM at pH 4.7 ± 0.1 under aerobiosis and anaerobiosis. Gentiobiose was supplemented into FCJM to 8.05 ± 0.1 mM. It is estimated that the gentiobiose source utilized contained at least 1.5 mM of the α‐anomer, melibiose. The colony counts from MRS and pH of FCJM at the end point are also presented

**Figure 6:**
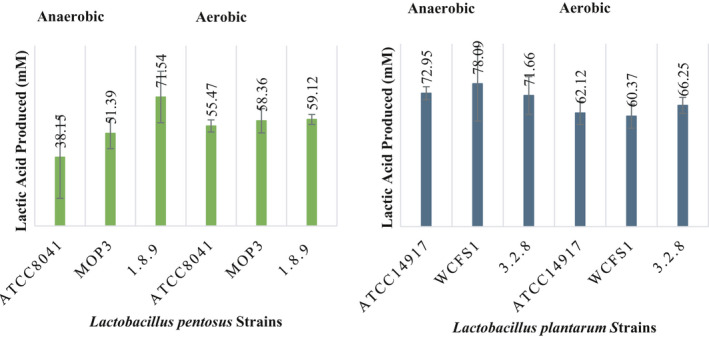
Production of lactic acid in FCJM supplemented with 8.05 ± 0.1 mM gentiobiose by single strains of Lactobacillus pentosus and *Lactobacillus plantarum*. The FCJM had a pH of 4.7 ± 0.1, and cultures were incubated aerobically and anaerobically. It is estimated that the gentiobiose source utilized contained at least 1.5 mM of the α‐anomer, melibiose. Colony counts from MRS and pH of FCJM at the end point are also presented

Figure 5 shows the utilization of 5.25 ± 0.59 and 4.45 ± 1.98 mM gentiobiose by *L. plantarum* and *L. pentosus*, respectively, from FCJM at pH 4.7 ± 0.1, regardless of oxygen availability and strain level differences. The *L. plantarum* and *L. pentosus* cultures produced 70.07 ± 7.56 and 55.69 ± 14.56 mM lactic acid (Figure 6), suggesting a strict homofermentation when gentiobiose was supplemented to lower amounts in the FCJM (8.05 ± 0.1 mM; Figure 5) as compared to the first experiment with 17.84 ± 3.14 mM (Figure 2). It was expected that approximately 32 mM lactic acid would be produced from the 8 mM gentiobiose supplemented, including the 1.5 mM of the anomeric impurity in the sugar source, melibiose. *L. plantarum* and *L. pentosus* produced 22.25 ± 13.40 and 14.6 ± 9.0 mM lactic acid, respectively in the un‐supplemented FCJM, raising the expected levels of lactic acid to 58 and 51 mM, respectively. As observed from the metabolism of cellobiose by *L. pentosus* and *L. plantarum* in FCJM at pH 4.7 ± 0.1, significant changes in pH to 3.82 ± 0.18 (*p* < .05) were observed after gentiobiose was utilized (Figure 5). The increases in cell densities ranged between 2 to 3 log CFU/ml as the result of gentiobiose utilization in FCJM at pH 4.7 ± 0.1 and were 1 log CFU/ml higher than those observed for cellobiose utilization under the same conditions (Figures 3 and 5).

In summary, LAB could utilize the disaccharides in cucumber fermentation, which become available as the result of tissue degradation due to spoilage or the enzymatic hydrolysis of cellulose. Despite some differences at the strain level, both *L. pentosus* and *L. plantarum*, which are the leading microbes in cucumber fermentations (Peréz‐Díaz et al., 2016), are capable of utilizing cellobiose and gentiobiose as an energy source at a growth permissive pH as a function of time. This observation is in line with previous reports in the literature as the ability of both of these bacterial species to utilize cellobiose and gentiobiose with some variations at the strain level (Fitzsimons et al., 1999; Siezen & van Hylckama Vlieg, 2011; Soltan Dallal et al., 2017; Sterr et al., 2009; Tamang et al., 2005; Yu et al., 2012). Variations in cellobiose utilization by *L. plantarum* strains were predicted by the bioinformatics analysis (Table 2). The inability to predict the same for the *L. pentosus* species may be associated with the fact that 107 *L. plantarum* genomes were used for the analysis instead of three genomes for the former.

## 4 CONCLUSIONS

The magnitude of the natural content of gentiobiose and cellobiose in fresh and fermented cucumbers and the utilization of the two disaccharides by certain LAB was determined. The plant‐derived disaccharides were utilized by *L. plantarum*, *L. pentosus,* and *L. buchneri* to variable extents in FCJM. *L. brevis* was unable to utilize cellobiose efficiently in FCJM. Cellobiose was homofermentatively utilized by LAB at pH 4.7 ± 0.1 but not at 3.7 ± 0.1. Supplementation of gentiobiose to FCJM at pH 4.7 to 8 and 18 mM resulted in homo‐ and heterofermentations, respectively. Some strain level differences were observed with regard to cellobiose utilization, but not in the conversion of gentiobiose. *L. plantarum* and *L. pentosus* were able to proliferate in FCJM at pH 4.7 in the absence of added energy sources and produced between 15 and 46 mM lactic acid. The ability of the LAB of relevance to cucumber fermentation to utilize the disaccharides may be of industrial concern, if the disaccharides become available from the degradation of cellulose by a postfermentation and prespoilage microbiota.

## AUTHOR CONTRIBUTIONS

Ms. R. A. Aslihan Ucar contributed the experimental designed and conducted the experiments, collected the data, interpreted the results, and drafted the manuscript. Dr. I. M. Pérez‐Díaz defined the scientific approach, contributed to the experimental design, and edited the manuscript. Dr. L. Dean contributed her analytical chemistry expertise, conducted the necessary analyses for the detection of cellobiose and gentiobiose in fresh, and fermented cucumbers and edited the manuscript.
